# Immune recognition of putative alien microbial structures: Host–pathogen interactions in the age of space travel

**DOI:** 10.1371/journal.ppat.1008153

**Published:** 2020-01-30

**Authors:** Mihai G. Netea, Jorge Domínguez-Andrés, Marc Eleveld, Huub J. M. op den Camp, Jos W. M. van der Meer, Neil A. R. Gow, Marien I. de Jonge

**Affiliations:** 1 Department of Internal Medicine and Radboud Center for Infectious Diseases, Radboud University Medical Center, Nijmegen, the Netherlands; 2 Department for Genomics & Immunoregulation, Life and Medical Sciences Institute (LIMES), University of Bonn, Bonn, Germany; 3 Department of Laboratory Medicine and Radboud Center for Infectious Diseases, Radboud University Medical Center, Nijmegen, the Netherlands; 4 Department of Microbiology, Faculty of Science, Radboud University, Nijmegen, the Netherlands; 5 School of Biosciences, University of Exeter, Exeter, United Kingdom; Vallabhbhai Patel Chest Institute, INDIA

## Abstract

Human space travel is on the verge of visiting Mars and, in the future, even more distant places in the solar system. These journeys will be also made by terrestrial microorganisms (hitchhiking on the bodies of astronauts or on scientific instruments) that, upon arrival, will come into contact with new planetary environments, despite the best measures to prevent contamination. These microorganisms could potentially adapt and grow in the new environments and subsequently recolonize and infect astronauts. An even more challenging situation would be if truly alien microorganisms will be present on these solar system bodies: What will be their pathogenic potential, and how would our immune host defenses react? It will be crucial to anticipate these situations and investigate how the immune system of humans might cope with modified terrestrial or alien microbes. We propose several scenarios that may be encountered and how to respond to these challenges.

## Background

The technological developments of the last half a century made space travel a reality. While the space race represented the quintessence of the competition between the United States and Soviet Union during the Cold War, the pace of space exploration subsequently decreased in the two decades after the fall of the Iron Curtain. This dynamic has changed dramatically in the last years, after the arrival on the scene of space exploration of new powers such as China, India, European Union, or Japan but especially since the dawn of commercial space flight, represented by companies such as SpaceX, Virgin Galactic, or Blue Origin. Space travel for humans is on the verge of changing from being the exclusive arena of a few elite astronauts and cosmonauts to a more egalitarian opportunity for entrepreneurs and wealthy adventurers. This new era in exploration of other planets such as Mars and the retrieval of physical samples invites speculation of measures that may become necessary to prevent biocontamination and biocontainment.

In parallel with the technological progress on Earth bringing human space travel closer to daily reality, a growing number of investigations have discovered places in the solar system on which life is possible and perhaps even probable [1]. The Apollo lunar missions and the Venera Soviet probes studying Venus have suggested it is unlikely that life will be found on our nearest neighboring moon or planet [2,3]. While this is generally also true for the studies looking for life on Mars, some ambiguity remains [4]. In the 1970s, the labeled release (LR) experiment conducted during the Viking mission, in which samples of Martian soil were inoculated with ^14^C-labeled microbial nutrients, resulted in an ambiguous outcome. The negative results of other experiments during this mission led to the final conclusion that “extant life was probably absent,” but the release of ^14^CO_2_ remained an intriguing finding [5]. Novel discoveries of the last two decades have increased again the hope for finding life outside Earth in the solar system. The likely presence of liquid water on Mars [6], the water ocean of Jupiter’s moon Europa [7], and the ocean-hosting volcanic vents on Saturn’s moon Enceladus [8] all suggest environments within the solar system much more conducive to life than we imagined a few years ago.

## Context

In the following years, humans will travel to other planets, and this time the aim will be more likely to reach Mars, rather than the sterile Moon. At the same time, long-range probes will be sent to Europa and other moons such as Enceladus or the carbohydrate-rich Titan satellite of the gas giant planets [9]. The journeys will inevitably be accompanied by terrestrial microorganisms hitchhiking on the bodies of the astronauts or on the scientific instruments. The human microbiota will, upon arrival, come into contact with new planetary environments, despite the best measures put into place to prevent contamination. These organisms have the potential to adapt and grow in the new environments and subsequently recolonize and infect astronauts and others upon return to Earth. It is important to anticipate this situation and investigate how the immune system of humans might react to this new reality.

In this perspective, we propose a number of research areas in “exo-immunology,” a term that we propose for a field concerned with the interaction between the immune system of terrestrial hosts and alien microbes or microorganisms derived from Earth that have adapted to extraterrestrial conditions.

## Are other solar system bodies habitable by terrestrial microorganisms?

Microbial life is found at practically every site on Earth and includes viruses, archaea, bacteria, fungi, unicellular plants, and protozoa. Even the most hostile locations on Earth have microbiological communities, and viable organisms have been revived from samples that date back thousands and even millions of years [10]. Being the simplest forms of life, the most likely form of exo-life on other solar system bodies will be probably equivalent to terrestrial bacteria or archaea. Such microorganisms are not only the most resilient and adaptable life forms known, but they are also the only known class of organisms that can survive independently, without reliance on other life forms. In addition, the putative microfossils present in the Allan Hills 84001 (ALH84001) meteorite are, if biotic, most likely bacterial [11].

Bacteria, by virtue of their normally haploid genomes and high growth rates, are amongst the most adaptable components of the microbiota. Some bacteria have a largely commensal ecology when growing in association with the human host, while others are obligate, facultative, or opportunistic pathogens [12]. An important characteristic of opportunistic pathogens is their capacity to adapt to a broad range of different habitats. These microorganisms are often nonfastidious or heterotrophic and can have only minimal nutritional requirements and are therefore able to survive and replicate in a range of different conditions [13]. Unicellular microorganisms are therefore likely to have the capacity to adapt quickly to extraterrestrial conditions. Equally, they will inevitably be carried by astronauts and/or equipment. This might, under favorable circumstances, lead to their colonization of new ecological niches on other planetary bodies.

The leading astrobiological theory is that life’s fundamental evolutionary nature has its origin in the abiotic cosmochemical evolution of biogenic elements [14]. It has been proposed that amino acids and organic compounds may have been deposited on Earth via the impact of comets, asteroids, and meteorites, where they promoted the origins of life through the provision of key molecular substrates. Carbon-containing meteorites have therefore been chemically analyzed, as insight into the composition of these objects will help to understand the biogenesis in our solar system [15].

The organic phase of carbonaceous meteorites is comprised of a complex mixture of largely uncharacterized solvent-extractable compounds, as well as soluble organic compounds such as amino acids, nucleobases, and polyhydroxylated substances or polyols, including sugars, sugar alcohols, and sugar acids [15]. Multiple mechanisms in the interstellar space and on the parent bodies of the carbonaceous meteorites (e.g., Murchison and Murray meteorites) may have contributed to the synthesis of soluble organic compounds. Photolysis of small molecules has been shown, by simulating interstellar conditions, to generate low molecular weight polyols [16,17]. In addition, condensation of formaldehyde via the formose reaction leads to the formation of carbohydrates in the interstellar space and in comets. This reaction might also have formed macromolecular carbon-containing compounds, part of which could be used as substrates for terrestrial bacteria.

This leads to the question of whether solar system bodies that are potential targets for mission landings might be habitable by terrestrial bacteria. This would be largely but not exclusively dependent on nutrient availability but also on the incidence of solar radiation, gravity, atmospheric pressure, magnetosphere, temperature, oxygen, and other gas concentrations and the availability of water. These complex circumstances will determine the chances of de novo colonization of these moons and planets. For example, changes in gravity induce morphogenetic changes in fungal hyphae [18], while exposure to galactic cosmic rays can cause a broad spectrum of mutations in *Bacillus subtilis* spores, which caused previously undescribed mutations in the bacterial genome related to resistance to antibiotics [19]. The analysis of gene expression of *Enterobacter bugandensis* isolated from the International Space Station showed an increase in the expression of genes involved in virulence, disease, and resistance to antibiotics compared to control strains [20]. On another note, some ancient terrestrial microorganisms such as Cyanobacteria can synthetize proteins to protect the bacterial DNA and rapidly recover from damage caused by space flight and ionizing radiation [21]. These mechanisms could be essential for the potential survival and adaptation of microorganisms to extraterrestrial environments.

Enceladus, a small, icy moon of Saturn, more than a billion kilometers further away from the Sun than Earth, was studied during the Cassini-Huygens National Aeronautics and Space Administration (NASA) Mission. Its average surface temperature reaches −198 °C, yet internal heat leads to the formation of geysers [22]. Measurements performed using a ion-neutral mass spectrometer (INMS) revealed that organic and nitrogen-containing molecules were released in the plume vapor, and the Cosmic Dust Analyzer (CDA) detected salts, strongly indicating a water ocean in contact with a core consisting of rocks [23,24]. Europa, one of the moons of Jupiter, is also covered with ice. The average surface temperature is −160 °C. The Hubble Space Telescope detected possible water vapor plumes on Europa’s surface due to an ocean of liquid water beneath its ice surface, with tidal heat allowing it to remain fluid [25].

Mars will be, most likely, the next space body to be visited by humans after the Moon. It has a thin atmosphere composed of 95% CO_2_, and there is evidence that liquid water could exist below the surface [26]. Mars lacks a magnetic field and has half the diameter of Earth, which consequently results in a less strong surface gravitational pull. The average surface temperature is −60 °C, but summertime temperatures can rise up to around 20 °C. This indicates potentially favorable conditions for the survival of terrestrial bacteria [27]. Therefore, at least three places outside Earth in our own solar system have environmental conditions that could be permissive for microbial life.

## What is the influence of space travel on the human immune system?

One of the most crucial concerns about human interplanetary exploration is the influence of the extreme conditions found in space on the human immune system. The experiences of nearly 600 astronauts who have been launched into space have shown that human beings can survive for several months in space without immediate detrimental effects on health [28]. Astronauts are exposed to harsh environmental conditions such as the influence of microgravity, solar radiation, variable magnetic fields, perturbations to normal circadian rhythms, restricted physical movement, poor nutritional intake, and psychological stress caused by isolation and physically constrained living environments. There is already evidence that astronauts experience altered immune function and increased vulnerability to infections during and after space flights. For example, almost 50% of the Apollo crew members suffered from either bacterial or viral infections during the mission or within seven days after their return to Earth [29].

Space travelers often experience reactivation of latent viral infections such as those caused by cytomegalovirus, varicella-zoster virus, and Epstein–Barr virus [30]. This reactivation of infection is associated with a rise in cortisol levels and a decrease in the production of interferon, which suggests a role for stress in the reactivation of these viral infections during spaceflight [31,32]. Astronauts experiencing extended spaceflights of at least six months had a general decay in T-cell function, which was accompanied by persistent reductions in the mitogen-stimulated production of cytokines such as interleukin (IL)-5, IL-6, IL-10, interferon gamma (IFNγ), and tumor necrosis factor alpha (TNFα) [33]. In this regard, flight conditions induced a decrease of IL-10 production in vitro after lipopolysaccharide (LPS) stimulation, whereas the concentrations of the neutrophil chemoattractant factor IL-8 were increased during space travel [33]. This increase coincides with the higher blood neutrophil cell numbers reported both after short and long flights, which increased up to 2-fold when compared to the initial counts [34,35] and the decreased numbers of precursors of monocytes and granulocytes in the bone marrow of astronauts [36,37].

The neutrophils and monocytes of astronauts tested postflight were found to exhibit reduced capacities of phagocytosis of bacteria and an attenuated oxidative burst and degranulation [38,39], suggesting a potentially decreased responsiveness for host defense cells against potential invading pathogens. When challenged with LPS, cosmonauts’ monocytes produced decreased amounts of the proinflammatory cytokines IL-6 and IL-1β and higher amounts of the antiinflammatory factor IL-1 receptor antagonist (IL-1Ra) compared to controls [35]. These changes progressively returned to baseline after return, with the exception of IL-1Ra, which remained five times higher in astronauts 6 to 12 months after landing. Regarding natural killer (NK) cells, exposure to microgravity conditions increased their apoptotic and necrotic activity concomitantly with a reduction in cell cytotoxicity, IFNγ, perforin expression, and delayed hypersensitivity responses [40,41].

In general, the investigations of the astronauts’ immune status revealed that there is a conversion from a situation of primed immune activity after acute gravitational stress to a progressive decay of the functionality of the immune system after several weeks of spaceflight. The risks associated with exposure to space environmental conditions or the lack of contact with terrestrial microbial stimuli increase with the amount of time spent in space. The complete absence of exposure to terrestrial antigens such as pollen, dust mite, environmental molds, or fungal spores during prolonged space expeditions would likely have an impact in the balance between T-helper and T-regulatory cells, which control the tolerance against self-antigens and innocuous environmental antigens [42].

These studies substantiate the view that the environmental insults that astronauts experience during space missions can compromise the function of their immune system, and therefore these conditions represent a considerable clinical hazard for astronauts undertaking extended journeys in space. However, only 24 astronauts have been beyond the low Earth orbit; thus the real consequences of longer term, deep-space travel for human immunity remain largely unknown.

In conclusion, the unpredictability in the behavior of the human immune system in space will only be reduced by improving our basic understanding of underlying biological processes of host defense during space travel. Only then, we will be able to develop appropriate strategies to ensure the safety and integrity of the space travelers in future missions.

## How would our immune host defenses react against “alien” and “modified” bacteria?

A second aspect of the consequences of space travel is to assess the pathogenic potential of putative alien microorganisms. This is likely to be determined by how efficient these microorganisms might grow in the environment of the human body and how the human immune system might respond.

**Fig 1:**
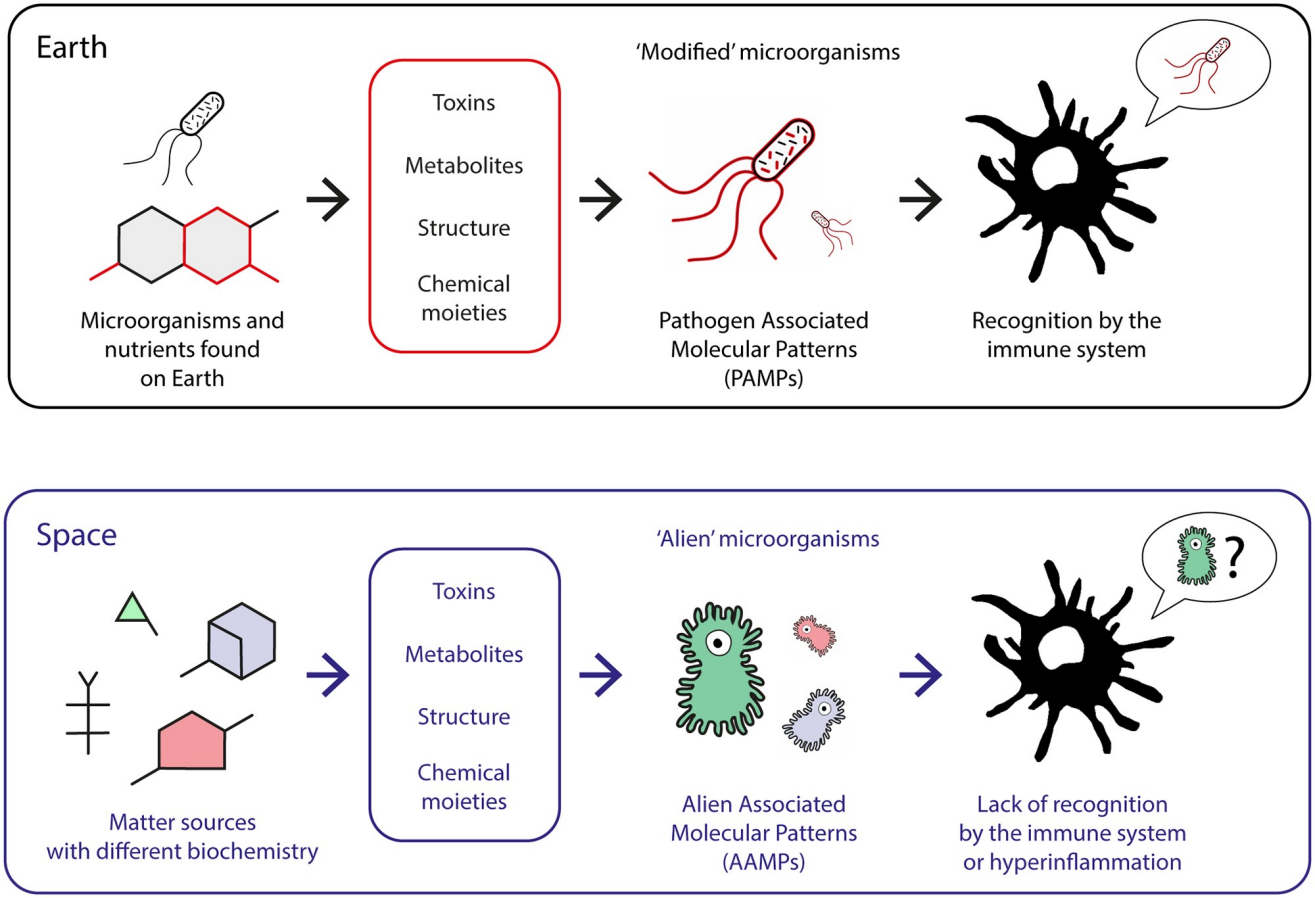
Interaction of the human immune system with alien microorganisms. In one scenario, the human immune system will interact with modified Earth microbes that have grown and adapted to an alien environment. In a second scenario, the human host defenses will encounter completely alien microbes, which may have different metabolic and cellular structures. It is a matter of speculation whether immune cells will be able to recognize such structures or, in contrast, to overreact to such an immune challenge.

The likelihood of an alien microbe having the ability to attach to cells of a terrestrial host and invade its cells or tissues, and hence produce infection, is obviously unknown. However, it is likely that alien microorganisms are adapted to entirely different conditions than those in terrestrial environments, are much less well adapted to the conditions of the human body, and will likely be less capable of colonization or invasion than our own microbiota. Even if such exomicrobes were not capable of directly invading the host and causing infection, they could still have pathogenic potential by secretion of toxins that could indirectly harm the astronauts (e.g., through wounds, contaminated food). Still, the majority of such toxins of terrestrial bacteria are proteins, which in turn are recognized by specific immune receptors (Fig 1). As amino acids are one of the most constant biochemical features present in nonterrestrial bodies, it may well be possible that such putative toxins of alien microbes are made of proteins.

**Fig 2:**
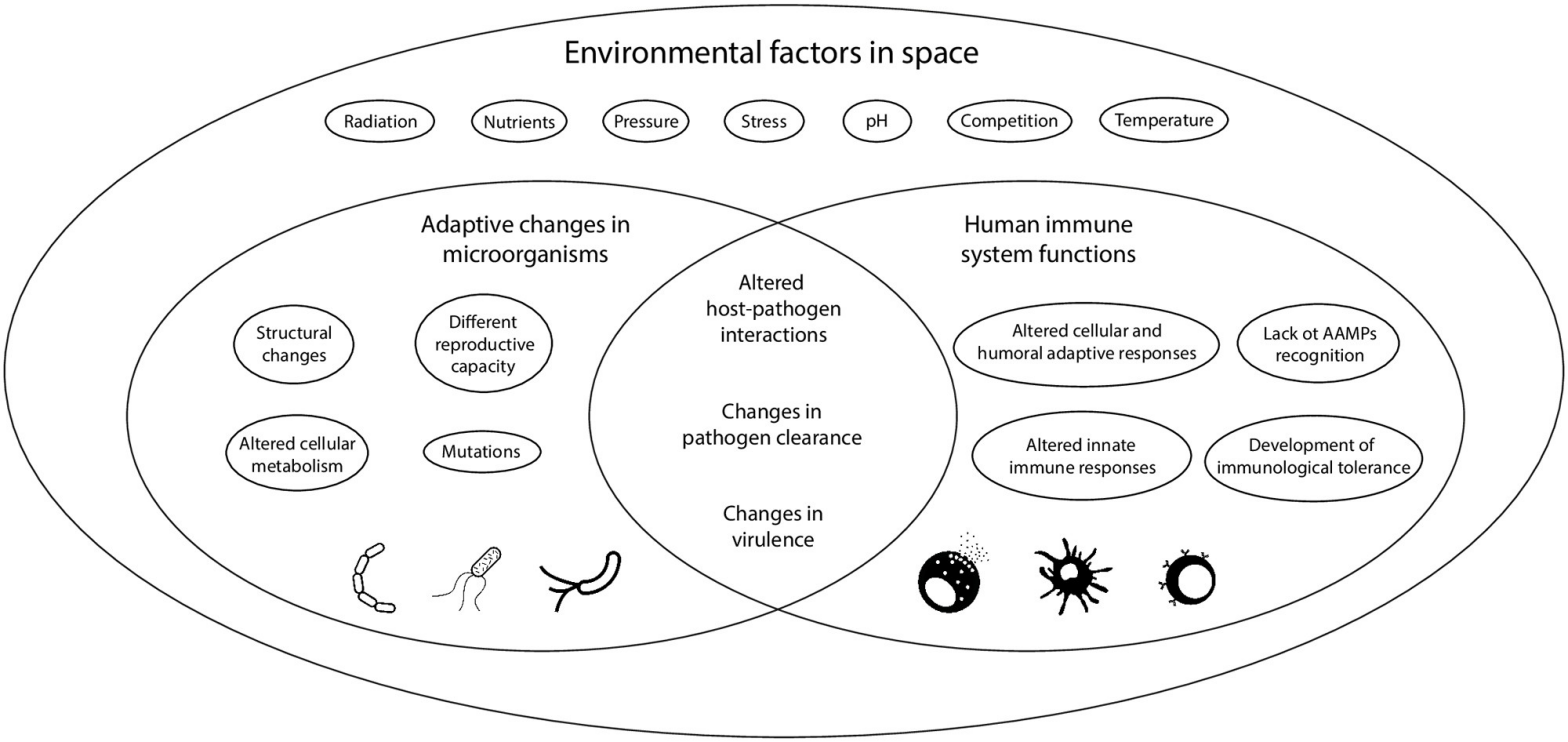
Interactions between modified Earth microorganisms and altered human immunity. Different space environmental factors that could impact on the one hand the microorganisms and on the other the immune system of the astronauts, leading to different outcomes of these host–pathogen interactions. AAMP, alien associated molecular patterns.

On the other hand, there is no doubt that modified microorganisms will be recognized by the human immune response leading to either an increased or decreased immune response, depending on the changes that the bacteria will undergo and the status of the host immune response during or after the space flight. Changes in human microbiome due to the conditions of space travel and adaptation of contaminating terrestrial pathogens to alien environments could also lead to the emergence of modified microorganisms with very different pathogenic potential (Fig 2).

## Conclusions and future perspectives

A new area of investigation for human microbiologists, immunologists, biochemists, and clinicians will likely emerge in the coming years. Humanity is at the cusp of extending direct space exploration involving human astronauts beyond Earth’s orbit and the Moon. The next steps that will be taken in this journey have the potential to bring us in contact with extraterrestrial life, most likely in a bacterial form. Even if these planetary bodies prove to be sterile, we can be sure that terrestrial microbes will travel together with human space explorers. These terrestrial microorganisms have the potential to adapt under the influence of novel evolutionary selective pressures on any exoplanet that is permissive to terrestrial microbial life. We need to anticipate and study the interactions between the terrestrial immune systems and alien or modified microbes in order to be prepared for these anticipated challenges.

## References

[1] Irwin LN, Schulze-Makuch D. Assessing the Plausibility of Life on Other Worlds. Astrobiology. Mary Ann Liebert, Inc.; 2001;1: 143–160. 10.1089/153110701753198918 12467118

[2] Margulis L, Guerrero R. Life as a planetary phenomenon: the colonization of Mars. Microbiologia. 1995;11: 173–84. 11539563

[3] Reese DE, Swan PR. Venera 4 Probes Atmosphere of Venus. Science (80-). 1968;159: 1228–1230. 10.1126/science.159.3820.1228 17814841

[4] Schulze-Makuch D, Irwin LN. Reassessing the Possibility of Life on Venus: Proposal for an Astrobiology Mission. Astrobiology. Mary Ann Liebert, Inc.; 2002;2: 197–202. 10.1089/15311070260192264 12469368

[5] Levin G V., Straat PA. The Case for Extant Life on Mars and Its Possible Detection by the Viking Labeled Release Experiment. Astrobiology. 2016;16: 798–810. 10.1089/ast.2015.1464 27626510 PMC6445182

[6] Orosei R, Lauro SE, Pettinelli E, Cicchetti A, Coradini M, Cosciotti B, et al Radar evidence of subglacial liquid water on Mars. Science (80-). 2018;361: eaar7268 10.1126/science.aar7268 30045881

[7] Carr MH, Belton MJS, Chapman CR, Davies ME, Geissler P, Greenberg R, et al Evidence for a subsurface ocean on Europa. Nature. 1998;391: 363–365. 10.1038/34857 9450749

[8] Parkinson CD, Liang M-C, Yung YL, Kirschivnk JL. Habitability of Enceladus: Planetary Conditions for Life. Orig Life Evol Biosph. 2008;38: 355–369. 10.1007/s11084-008-9135-4 18566911

[9] Prangé R, Pallier L, Hansen KC, Howard R, Vourlidas A, Courtin R, et al An interplanetary shock traced by planetary auroral storms from the Sun to Saturn. Nature. 2004;432: 78–81. 10.1038/nature02986 15525983

[10] Shen-Miller J, Schopf JW, Harbottle G, Cao R -j., Ouyang S, Zhou K -s., et al Long-living lotus: germination and soil -irradiation of centuries-old fruits, and cultivation, growth, and phenotypic abnormalities of offspring. Am J Bot. 2002;89: 236–247. 10.3732/ajb.89.2.236 21669732

[11] Gibson EK, McKay DS, Thomas-Keprta KL, Wentworth SJ, Westall F, Steele A, et al Life on Mars: evaluation of the evidence within Martian meteorites ALH84001, Nakhla, and Shergotty. Precambrian Res. Elsevier; 2001;106: 15–34. 10.1016/S0301-9268(00)00122-4

[12] Raffatellu M. Learning from bacterial competition in the host to develop antimicrobials. Nat Med. 2018;24: 1097–1103. 10.1038/s41591-018-0145-0 30082869

[13] Zarecki R, Oberhardt MA, Reshef L, Gophna U, Ruppin E. A novel nutritional predictor links microbial fastidiousness with lowered ubiquity, growth rate, and cooperativeness. PLoS Comput Biol. Public Library of Science; 2014;10: e1003726 10.1371/journal.pcbi.1003726 25033033 PMC4102436

[14] Lazcano A. Historical Development of Origins Research. Cold Spring Harb Perspect Biol. 2010;2: a002089–a002089. 10.1101/cshperspect.a002089 20534710 PMC2964185

[15] Pizzarello S, Shock E. Carbonaceous Chondrite Meteorites: the Chronicle of a Potential Evolutionary Path between Stars and Life. Orig Life Evol Biosph. 2017;47: 249–260. 10.1007/s11084-016-9530-1 28078499

[16] Khare BN, Thompson WR, Chyba CF, Arakawa ET, Sagan C. Organic solids produced from simple C/H/O/N ices by charged particles: applications to the outer solar system. Adv Space Res. 1989;9: 41–53. 10.1016/0273-1177(89)90362-1 11537360

[17] Agarwal VK, Schutte W, Greenberg JM, Ferris JP, Briggs R, Connor S, et al Photochemical reactions in interstellar grains photolysis of CO, NH3, and H2O. Orig Life Evol Biosph. 1985;16: 21–40. 10.1007/bf01808047 11542015

[18] Moore D. Graviresponses in fungi. Adv Sp Res. 1996;17: 73–82. 10.1016/0273-1177(95)00614-K 11538639

[19] Moeller R, Reitz G, Berger T, Okayasu R, Nicholson WL, Horneck G. Astrobiological Aspects of the Mutagenesis of Cosmic Radiation on Bacterial Spores. Astrobiology. 2010;10: 509–521. 10.1089/ast.2009.0429 20624059

[20] Singh NK, Bezdan D, Checinska Sielaff A, Wheeler K, Mason CE, Venkateswaran K. Multi-drug resistant Enterobacter bugandensis species isolated from the International Space Station and comparative genomic analyses with human pathogenic strains. BMC Microbiol. BioMed Central; 2018;18: 175 10.1186/s12866-018-1325-2 30466389 PMC6251167

[21] Klementiev KE, Maksimov EG, Gvozdev DA, Tsoraev GV., Protopopov FF, Elanskaya I V., et al Radioprotective role of cyanobacterial phycobilisomes. Biochim Biophys Acta—Bioenerg. 2019;1860: 121–128. 10.1016/j.bbabio.2018.11.018 30465750

[22] Waite JH, Combi MR, Ip W-H, Cravens TE, McNutt RL, Kasprzak W, et al Cassini ion and neutral mass spectrometer: Enceladus plume composition and structure. Science. American Association for the Advancement of Science; 2006;311: 1419–22. 10.1126/science.1121290 16527970

[23] Waite JH Jr, Lewis WS, Magee BA, Lunine JI, McKinnon WB, Glein CR, et al Liquid water on Enceladus from observations of ammonia and 40Ar in the plume. Nature. 2009;460: 487–490. 10.1038/nature08153

[24] Postberg F, Schmidt J, Hillier J, Kempf S, Srama R. A salt-water reservoir as the source of a compositionally stratified plume on Enceladus. Nature. Nature Publishing Group; 2011;474: 620–622. 10.1038/nature10175 21697830

[25] Roberts JH, Nimmo F. Tidal heating and the long-term stability of a subsurface ocean on Enceladus. Icarus. 2008;194: 675–689. 10.1016/j.icarus.2007.11.010

[26] Jones EG. Shallow transient liquid water environments on present-day mars, and their implications for life. Acta Astronaut. 2018;146: 144–150. 10.1016/j.actaastro.2018.02.027

[27] Cabrol NA. The Coevolution of Life and Environment on Mars: An Ecosystem Perspective on the Robotic Exploration of Biosignatures. Astrobiology. 2018;18: 1–27. 10.1089/ast.2017.1756 29252008 PMC5779243

[28] Stepanek J, Blue RS, Parazynski S. Space Medicine in the Era of Civilian Spaceflight. Longo DL, editor. N Engl J Med. 2019;380: 1053–1060. 10.1056/NEJMra1609012 30865799

[29] Taylor GR, Graves RC, Brockett RM, Ferguson JK, Mieszkuc BJ. Skylab environmental and crew microbiology studies. 1977; https://ntrs.nasa.gov/search.jsp?R=19770026844

[30] Rooney B V., Crucian BE, Pierson DL, Laudenslager ML, Mehta SK. Herpes Virus Reactivation in Astronauts During Spaceflight and Its Application on Earth. Front Microbiol. Frontiers; 2019;10: 16 10.3389/fmicb.2019.00016 30792698 PMC6374706

[31] Cohrs RJ, Mehta SK, Schmid DS, Gilden DH, Pierson DL. Asymptomatic reactivation and shed of infectious varicella zoster virus in astronauts. J Med Virol. 2008;80: 1116–1122. 10.1002/jmv.21173 18428120 PMC2938738

[32] Mehta SK, Tyring SK, Cohrs RJ, Gilden D, Feiveson AH, Lechler KJ, et al Rapid and sensitive detection of varicella zoster virus in saliva of patients with herpes zoster. J Virol Methods. NIH Public Access; 2013;193: 128 10.1016/J.JVIROMET.2013.05.019 23747545 PMC3735804

[33] Crucian B, Stowe RP, Mehta S, Quiriarte H, Pierson D, Sams C. Alterations in adaptive immunity persist during long-duration spaceflight. npj Microgravity. 2015;1: 15013 10.1038/npjmgrav.2015.13 28725716 PMC5515498

[34] Stowe RP, Sams CF, Mehta SK, Kaur I, Jones ML, Feeback DL, et al Leukocyte subsets and neutrophil function after short-term spaceflight. J Leukoc Biol. 1999;65: 179–86. 10.1002/jlb.65.2.179 10088600

[35] Kaur I, Simons ER, Kapadia AS, Ott CM, Pierson DL. Effect of spaceflight on ability of monocytes to respond to endotoxins of gram-negative bacteria. Clin Vaccine Immunol. American Society for Microbiology; 2008;15: 1523–8. 10.1128/CVI.00065-08 18768671 PMC2565938

[36] Sonnenfeld G, Mandel AD, Konstantinova IV., Berry WD, Taylor GR, Lesnyak AT, et al Spaceflight alters immune cell function and distribution. J Appl Physiol. 1992;73: S191–S195. 10.1152/jappl.1992.73.2.S191 1526951

[37] Ichiki AT, Gibson LA, Jago TL, Strickland KM, Johnson DL, Lange RD, et al Effects of spaceflight on rat peripheral blood leukocytes and bone marrow progenitor cells. J Leukoc Biol. 1996;60: 37–43. 10.1002/jlb.60.1.37 8699121

[38] Kaur I, Simons ER, Castro VA, Ott CM, Pierson DL. Changes in monocyte functions of astronauts. Brain Behav Immun. 2005;19: 547–554. 10.1016/j.bbi.2004.12.006 15908177

[39] Kaur I, Simons ER, Castro VA, Mark Ott C, Pierson DL. Changes in neutrophil functions in astronauts. Brain Behav Immun. 2004;18: 443–450. 10.1016/j.bbi.2003.10.005 15265537

[40] Li Q, Mei Q, Huyan T, Xie L, Che S, Yang H, et al Effects of simulated microgravity on primary human NK cells. Astrobiology. Mary Ann Liebert, Inc.; 2013;13: 703–14. 10.1089/ast.2013.0981 23919749 PMC3746215

[41] Guéguinou N, Huin-Schohn C, Bascove M, Bueb J-L, Tschirhart E, Legrand-Frossi C, et al Could spaceflight-associated immune system weakening preclude the expansion of human presence beyond Earth’s orbit? J Leukoc Biol. Wiley-Blackwell; 2009;86: 1027–1038. 10.1189/jlb.0309167 19690292

[42] Bacher P, Heinrich F, Stervbo U, Nienen M, Vahldieck M, Iwert C, et al Regulatory T Cell Specificity Directs Tolerance versus Allergy against Aeroantigens in Humans. Cell. 2016;167: 1067–1078.e16. 10.1016/j.cell.2016.09.050 27773482

